# Rescuing the Golgi from heat damages by ATG8: restoration rather than clean-up

**DOI:** 10.1007/s44154-023-00100-6

**Published:** 2023-06-26

**Authors:** Anni Luo, Jian-Xiang Liu

**Affiliations:** State Key Laboratory of Plant Physiology and Biochemistry, College of Life Sciences, Zhejiang University, Hangzhou, 310027 China

**Keywords:** ATG8, Autophagy, CLC2, ER, Golgi restoration, Heat stress

## Abstract

High temperature stress poses significant adverse effects on crop yield and quality. Yet the molecular mechanisms underlying heat stress tolerance in plants/crops, especially regarding the organellar remodeling and homeostasis, are largely unknown. In a recent study, Zhou et al. reported that autophagy-related 8 (ATG8), a famous regulator involved in autophagy, plays a new role in Golgi restoration upon heat stress. Golgi apparatus is vacuolated following short-term acute heat stress, and ATG8 is translocated to the dilated Golgi membrane and interacts with CLATHRIN LIGHT CHAIN 2 (CLC2) to facilitate Golgi restoration, which is dependent on the ATG conjugation system, but not of the upstream autophagic initiators. These exciting findings broaden the fundamental role of ATG8, and elucidate the organelle-level restoration mechanism of Golgi upon heat stress in plants.

## Introduction

Heat stress (HS) affects plant growth and development, leading to a decrease in yield and quality (Li et al. 2023). With the global warming, it has become pressing to investigate the adaptive mechanisms of plants to high temperature stress. As sessile organisms, plants have evolved sophistic mechanisms to acclimate temperature changes, such as heat sensing, signal transduction, membrane fluidity modification and downstream responses (Zhang et al. 2019). Under HS, there are structural modifications in the cytoskeleton and organelles of plant cells, which in turn alter the functionality of the cell membrane system. The denaturation of proteins in endoplasmic reticulum (ER) is the most detrimental effect of heat stress, which disrupts the homeostasis of intracellular proteins (Liu et al. 2020; Sun et al. 2021). Apart from ER, other organelles, such as chloroplast, mitochondrion, peroxisome, nucleus, and cell wall, are associated with ambient temperature stress, which would generate stress signals combined to regulate the expression of stress-responsive genes and other cellular processes to restore cellular homeostasis (Zhu 2016). Recently, QTL analysis of heat stress tolerance identifies a RING-type E3 ligase Thermo-tolerance 3.1 (TT3.1) that relocates from plasma membrane to endosomes to degrade TT3.2 for protecting chloroplasts in response to heat stress in rice (Li et al. 2023; Zhang et al. 2022). Besides, Golgi is important for the activation of ER stress responses upon HS (Che et al. 2010; Sun et al. 2013, 2015). HS also causes disorganization of the Golgi apparatus, but the restoration mechanism in plants is largely unknown.

Autophagy is an adaptive response strategy for plants to cope with heat stress, which specifically identifies and degrades damaged organelles as a quality control mechanism (Sun et al. 2021). There are several core ATG proteins involved in autophagosome formation, which can be divided into five groups: the ATG1 complex, the PI3K complex, the ATG2–ATG18 complex, the ATG9 vesicles and the ATG conjugation systems (Han et al. 2011). ATG8 is part of the ATG conjugation systems, which bind to autophagosome membranes to facilitate autophagosome biogenesis and selective cargo degradation (Bu et al. 2020). Surprisingly, a recent study conducted by Zhou et al. reports a novel role of Arabidopsis ATG8 in Golgi restoration upon HS, which is not dependent on the upstream autophagic initiators (Zhou et al. 2023). These exciting findings expand the basic role of ATG8, and reveal the restoration mechanism of Golgi upon HS at organellar level.

When plants were exposed to a short period of high temperature (45 ºC), all nine ATG8 isoforms (ATG8a to ATG8i) were seen to form punctate structures in wild-type plants, in contrast, the formation of GFP-ATG8a punctate structures was not observed in *atg5-1*, *atg7-2* and *atg16-c1* mutants lacking key components of the conventional ATG conjugation system (Zhou et al. 2023). These results suggest that ATG8 puncta formation relies on the ATG conjugation system instead of other complexes involved in the early step of autophagy. Zhou et al. later found that upon HS, the majority of mCherry–ATG8f puncta did not overlap with NBR1–GFP, an autophagic receptor (Zhou et al. 2023), indicating HS-induced ATG8 puncta may be involved in an alternative pathway of cellular stress response that was distinct from the conventional autophagy pathway. Coincidentally, ATG8 interacts with ABNORMAL SHOOT3 (ABS3) subfamily of multidrug and toxic compound extrusion (MATE) transporters at the late endosome to promote senescence and protein degradation in Arabidopsis without canonical cleavage and lipidation of ATG8 (Jia et al. 2019).

Electron microscopy analysis further showed that ATG8 puncta colocalized with swollen Golgi stacks upon HS, while other ATG conjugation components were not noticeably recruited to the Golgi apparatus (Zhou et al. 2023). These results suggest ATG8 relocates to Golgi membranes. Time-resolved electron tomography was performed to study the morphological transitions during Golgi restoration from swollen cisternae. They discovered that the swollen cisternae and the disruption of the Golgi stack architecture occurred immediately after 5 min of HS treatment (Zhou et al. 2023). Root cells of wild-type plants had typical stacked Golgi after 6 h of recovery from HS, however, in *atg5* mutant plants, there was no stacked Golgi at 6 h of recovery, and disk-shaped cisternae were recognized only after 12 h recovery (Zhou et al. 2023). These results suggest that HS-induced swollen cisternae of Golgi are reversible after HS recovery, and disruption of ATG5 delays the restoration process.

Since ATG8 is essential for the recovery of Golgi stacks, it prompts the authors to identify proteins associated with ATG8 upon HS. With the help of TurboID-mediated proximity labelling proteomics of ATG8, they identified overlapping proteins of HS-treated TurboID–YFP–ATG8a (TI–Y8a) versus HS-treated TurboID–YFP (TI–YFP) group and HS-treated TI–Y8a versus HS-untreated TI–Y8a group. The result showed that 6 proteins were not only significantly enriched in the Golgi/TGN-related cellular components, but also associated with clathrins (Zhou et al. 2023). Among them, CLATHRIN LIGHT CHAIN 2 (CLC2) interacted with ATG8 in Y2H assays, BiFC assays and Co-IP assays (Zhou et al. 2023). They also confirmed the AIM (ATG8 interact motif) site of CLC2 and the docking site (LDS) of ATG8e were required for the interaction in Y2H assays and in vivo recruitment assays (Zhou et al. 2023).

The colocalization between GFP–ATG8a and CLC2–mCherry was markedly enhanced upon HS in root cells of double overexpression transgenic plants (Zhou et al. 2023). Consistently, HS increased the colocalization of CLC2–mCherry with not only VHA-a1-YFP (the TGN marker) but also GFP-SYP32 (the cis-Golgi marker), while in the *atg5-1* mutant such colocalization was notably reduced (Zhou et al. 2023). Nevertheless, mutation of *CLC2* did not affect the formation of YFP–ATG8e puncta after HS treatment (Zhou et al. 2023). These results imply that CLC2 is recruited to the swollen Golgi by ATG8 under HS treatment. They later found Golgi recovery was delayed in *clc2-1* mutant, indicating that CLC2 is involved in restoration of the Golgi/TGN complex (Zhou et al. 2023). To investigate the dynamics of ATG8-positive vesicles, they conducted long-term time-lapse imaging analysis. The results confirmed that with wortmannin (an inhibitor of PI3K) treatment that accelerates vacuole fusion, the signals of mCherry–ATG8f were significantly observed on the vacuole membranes (Zhou et al. 2023). These findings suggest that the vesicular cisternae budded ATG8-positive vesicles may eventually merge with vacuole membranes, which facilitates clearing the blocked cargoes accumulated in the swollen Golgi, leading to quick restoration of Golgi stacks. Although CLC2 has been confirmed to participate in the recovery of HS-induced swollen Golgi, the exact mechanism on how CLC2- or clathrin-mediated budding contributes to Golgi reorganization is not known.

In summary, Zhou et al. (2023) revealed a noncanonical role of ATG8 in Golgi remodeling upon HS in Arabidopsis. Specifically, HS causes vesiculation of the Golgi at the organelle level, and ATG8 is relocated to Golgi membranes to assist in Golgi restoration together with its interactor CLC2. In future studies, it would be interesting to investigate the coordination between ER-to-Golgi transport and ATG8-positive vesicles in Golgi remodeling, which is important for developing heat resilient plants. Considering that plants are confronted with various types of stress, it would be worthwhile to investigate whether ATG8 has noncanonical roles beyond heat stress.

## Data Availability

Not applicable.

## Abbreviations

AIM: ATG8 interact motif
ATG8: Autophagy-related 8
CLC2: CLATHRIN LIGHT CHAIN 2
ER: Endoplasmic reticulum
HS: Heat stress
TT3.1: Thermo-tolerance 3.1
